# Artificial intelligence in tissue engineering and regenerative medicine: from algorithmic capability to intelligent integration

**DOI:** 10.3389/fbioe.2026.1933082

**Published:** 2026-09-23

**Authors:** Melanie L. Hart, Mary C. Walsh, Corinna Raimondo, Ryuji Kato, Bernd Rolauffs

**Affiliations:** 1 G.E.R.N. Research Center for Tissue Replacement, Regeneration and Neogenesis, Department of Orthopedics and Trauma Surgery, Faculty of Medicine, Medical Center-Albert-Ludwigs-University of Freiburg, Freiburg im Breisgau, Germany; 2 Maidstone Consulting Group, Boston, MA, United States; 3 Graduate School of Pharmaceutical Sciences, Nagoya University, Tokai National Higher Education and Research System, Nagoya, Aichi, Japan

**Keywords:** artificial intelligence, generative AI, human oversight, large language models, regenerative medicine, responsible AI, risks, tissue engineering

## Abstract

Artificial intelligence (AI) is increasingly integrated throughout the tissue engineering and regenerative medicine (TERM) pipeline, from literature synthesis, experimental design, and automation to image analysis, biological modeling, and knowledge dissemination. However, TERM differs fundamentally from many domains where AI has succeeded. Biological heterogeneity, limited datasets, evolving protocols, sparse ground truth, and high translational stakes constrain the development and deployment of AI-driven approaches. Consequently, AI should be seen as a collection of purpose-specific tools whose suitability, risks, and validation requirements depend on the context. This review examines how AI can be intelligently integrated across the TERM workflow, emphasizing how intended purpose, biological reality, and translational constraints shape technology selection. We examine AI’s use as a framework for knowledge extraction, experimental planning, decision support, experimental execution, data generation, interpretation, and scientific communication. Key challenges include limited generalizability, uncertainty, validation, accountability, and the risk of confusing predictive performance with biological or mechanistic insight. We argue that many important gaps in AI-enabled TERM are not algorithmic but conceptual, methodological, and educational. Future progress will depend on purpose-aware AI literacy, uncertainty-aware modeling, validation strategies, human-in-the-loop workflows, and establishing community standards for transparency, reproducibility, and translation. Ultimately, AI should not be a shortcut around biological complexity, but a means of engaging with that complexity more explicitly and responsibly. Across the TERM pipeline, AI is best viewed as an amplifier of biological evidence and scientific reasoning rather than a substitute, with its greatest impact arising from intelligent integration into scientific practice rather than increasingly intelligent algorithms alone.

## 1 Introduction: AI in tissue engineering and regenerative medicine: promise, constraints, and translational realities

Tissue engineering and regenerative medicine (TERM) are advancing rapidly, driven by breakthroughs in biomaterials, cell fate engineering, biofabrication, and high-resolution analytical tools. Together, these developments have increased the complexity of data generated across the field (Balkhair and Albalushi, 2025; Kim et al., 2026; Moroni et al., 2018; Song et al., 2025). In TERM, artificial intelligence (AI) technologies, including machine learning, computer vision, and large language models (LLMs), are increasingly used to streamline scientific drafting, accelerate scientific discovery, optimize experimental design, uncover biological patterns, and support translational decision-making (Guo et al., 2023; Nosrati and Nosrati, 2023; Asadi Sarabi et al., 2024; Gharibshahian et al., 2024; Rolauffs et al., 2026). Across these diverse applications, AI could be viewed as a technology for augmenting human scientific judgment rather than replacing it. Because AI comprises multiple technologies used at different stages of research, we use “AI” as an umbrella term encompassing machine learning, deep learning, computer vision, generative AI, LLMs, active learning, optimization algorithms, and hybrid mechanistic–data-driven models (Guo et al., 2023; Nosrati and Nosrati, 2023; Waters et al., 2021; Rolauffs et al., 2026). Meaningful integration must therefore consider the application domain, intended purpose, and technology type, consistent with the Organisation for Economic Co-operation and Development (OECD) framework (OECD, 2022).

Unlike data-rich, low-risk domains where AI has succeeded through stable labels and abundant standardized datasets, TERM involves substantial biological heterogeneity, limited samples, evolving experimental protocols, sparse ground truth, high translational stakes, and criteria that shift across *in vitro*, *ex vivo*, *in vivo*, and clinical contexts. These constraints shape which AI technologies are feasible, reliable, and clinically meaningful. Thus, AI serves as a lens to examine how complexity and scarcity shape methodological choices in TERM. Particular emphasis is placed not only on technical capability, but also on reproducibility, uncertainty, robustness, interpretability, and responsible integration into experimentally and clinically constrained environments. The central challenge is incorporating AI into TERM workflows to strengthen experimental rigor and reduce translational risk.

Targeted literature searches were conducted between April and July 2026 using PubMed/MEDLINE and Web of Science, supplemented by relevant journal websites. Claude Sonnet 5 and Claude Opus 4.8 (Anthropic) were additionally used to support literature identification; all AI-suggested sources were independently verified by the authors for accuracy and relevance before inclusion (see AI Statement). Authors identified literature within their respective fields of expertise and collectively integrated the selected sources into the five-stage TERM framework.

## 2 Context matters: defining TERM-specific constraints for AI

Several core constraints distinguish TERM from many other application domains of AI:

- Data scarcity and heterogeneity: experiments often rely on limited numbers of donors, animals, engineered constructs, or patient-derived samples, while substantial biological heterogeneity and high experimental costs restrict large-scale dataset generation and reproducibility (Ahmed et al., 2024; Gharibshahian et al., 2024; Guo et al., 2023; Jeznach et al., 2024; Plant et al., 2022; Ramesh et al., 2024; Waters et al., 2021; Zhang et al., 2025).
- Workflow dependency: differences in sample handling, scaffold fabrication, biofabrication parameters, culture conditions, imaging systems, and analysis pipelines can strongly influence experimental outcomes and introduce substantial technical variability (Ahmed et al., 2024; Kim et al., 2026; Plant et al., 2022; Zhang et al., 2025)
- Sparse ground truth: biological systems are inherently noisy and dynamic, while labels such as tissue quality, maturation, regeneration, or functional success are often implicit or provisional (Ahmed et al., 2024; Guo et al., 2023; Klabukov et al., 2026; Waters et al., 2021; Zhang et al., 2025).
- Contextual instability: outcome measures and definitions of biological success are continuously refined as scientific understanding evolves and these measures can shift across *in vitro*, *in vivo*, and clinical settings, complicating model validation, generalizability, and translational interpretation (Klabukov et al., 2026; Plant et al., 2022; Waters et al., 2021).
- High translational stakes: in TERM, clinical benefit, safety, reproducibility, traceability, and regulatory compliance outweigh technical novelty, and experimental or predictive errors may carry significant ethical and translational consequences (Balkhair and Albalushi, 2025; Garmany and Terzic, 2024; Nosrati and Nosrati, 2023; Plant et al., 2022; Waters et al., 2021).

These constraints mean AI should be evaluated not by predictive performance alone, but by its ability to generate biologically meaningful insights and support robust experimental and translational decisions (Plant et al., 2022; Waters et al., 2021). This context-of-use approach is consistent with credibility and regulatory frameworks from ASME, EMA, and the FDA (American Society of Mechanical Engineers, 2018; European Medicines Agency, 2024; U.S. Food and Drug Administration, 2023; U.S. Food and Drug Administration, 2025a; U.S. Food and Drug Administration, 2025b; U.S. Food and Drug Administration et al., 2021).

## 3 How AI is used along the TERM pipeline: purposes, technologies, and risks

AI serves diverse functions across the TERM pipeline, categorized into five primary roles: (1) knowledge extraction, (2) design and decision support, (3) experimental execution, (4) data interpretation, and (5) scientific communication. As illustrated in Figure 1, AI integration depends on the intended purpose, the technology class employed, and the associated risks and validation needs. Furthermore, this pipeline is not linear but iterative; for instance, data generated during experimental execution (Stage 3) provides critical feedback to the knowledge extraction and planning phase (Stage 1), enabling the iterative refinement of original hypotheses based on real-world biological evidence. Across all stages, AI functions as an amplifier of scientific reasoning and evidence generation, while human accountability remains paramount (Table 1).

**FIGURE 1:**
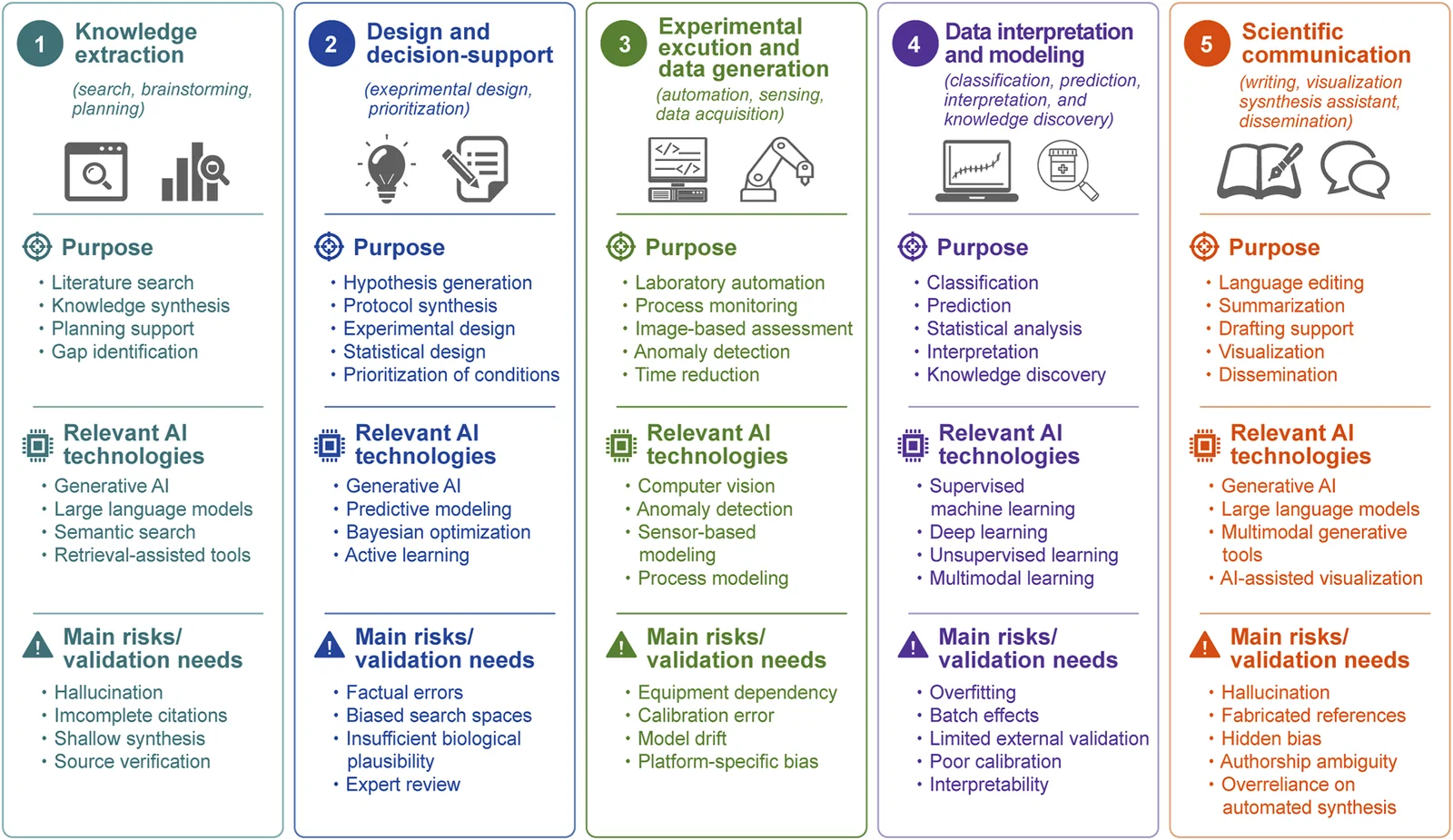
Mapping AI use across the TERM pipeline by purpose, technology, and risk.

**TABLE 1:** Human responsibilities and oversight requirements across the TERM pipeline.

TERM stage	Primary human responsibility	Desired human oversight
Knowledge extraction	Critical appraisal and evidence selection	Verification of sources and relevance determination
Design and decision-support	Scientific reasoning and experimental prioritization	Expert evaluation of biological plausibility and experimental feasibility
Experimental execution and data generation	Experimental observation and process control	Experimental validation, calibration, and intervention
Data interpretation and modeling	Biological interpretation	External validation uncertainty, and limitations assessments
Scientific communication	Scientific accountability	Author verification, disclosure, and responsibility

### 3.1 Knowledge extraction (Stage 1)

The initial stage of the TERM pipeline focuses on the efficient synthesis of existing scientific data and the identification of research gaps. For these tasks, the workflow relies primarily on generative AI, LLMs, and retrieval-assisted tools (Table 2). These technologies accelerate the process of literature search and planning support, allowing researchers to navigate vast amounts of data more rapidly. However, the utility of these systems is constrained by risks such as hallucinations, incomplete and fabricated citations, shallow synthesis, and the reproduction of existing biases (Cirillo et al., 2020; Ferrara, 2023; van Dis et al., 2023; Resnik and Hosseini, 2026; Topaz et al., 2026). To mitigate these, researchers can employ structured prompt engineering using explicit task definitions, inclusion/exclusion criteria, and iterative refinement to ensure more rigorous, researcher-guided knowledge synthesis (Vilakati, 2025; Ng, 2026). Consequently, these tools should be used as a framework for knowledge discovery rather than as an authoritative source, necessitating rigorous source verification and critical human appraisal to ensure scientific accuracy. In this view, effective AI use begins with asking scientifically meaningful questions rather than merely engineering technically sophisticated prompts to obtain plausible answers.

**TABLE 2:** Summary of AI technology integration across the TERM pipeline.

TERM stage	Relevant AI technologies	Primary purposes and use cases	Key strengths	Main risks and validation needs	Specific TERM examples
1. Knowledge extraction	Generative AI; LLMs; semantic search; retrieval-assisted tools	Literature search; knowledge synthesis; planning support; gap identification	Rapid synthesis of vast literature; efficient gap identification	Hallucination; incomplete or fabricated citations; shallow synthesis; need for source verification	Literature synthesis and gap identification (Cirillo et al., 2020; Ferrara, 2023; Ng, 2026; van Dis et al., 2023; Resnik and Hosseini, 2026; Topaz et al., 2026)
2. Design and decision support	Generative AI; predictive modeling; Bayesian optimization; active learning	Hypothesis generation; protocol synthesis; experimental design; statistical design; prioritization of conditions	Reduction of combinatorial experimental burden; data-driven design optimization	Factual errors; biased search spaces; insufficient biological plausibility; need for expert review	Material characteristics, including composition, pore geometry, and degradation kinetics (Ahmed et al., 2024; Guo et al., 2023; Liu et al., 2025); biofabrication printability, structural fidelity, and parameter tuning (Ramesh et al., 2024; Wu et al., 2024; Zhang et al., 2025)
3. Experimental execution and data generation	Computer vision; anomaly detection; sensor-based modeling; process modeling	Laboratory automation; process monitoring; image generation; image-based assessment; anomaly detection; reduced acquisition and processing time	Real-time quality control; increased precision in data acquisition	Equipment dependency; calibration error; model drift; platform-specific bias	Cell counting; segmentation; morphological analysis; organoid monitoring (Balkhair and Albalushi, 2025; Jeznach et al., 2024); robotic handling systems; protocol optimization; early detection of failure modes; bioreactor control (pH, oxygen, nutrient delivery) (Banerejee and Lahiri, 2026; Helleckes et al., 2026)
4. Data interpretation and modeling	Supervised ML; deep learning; unsupervised learning; multimodal learning	Classification; prediction; statistical analysis; interpretation; knowledge discovery	Pattern recognition in high-dimensional data; predictive modeling of outcomes	Overfitting; batch effects; limited external validation; poor calibration; limited interpretability	Omics preprocessing (noise reduction, clustering, feature extraction, and batch correction); image classification; segmentation; cell fate/phenotype classification; integration of multimodal omics (transcriptomics, proteomics, metabolomics, and spatial biology) (Ahmed et al., 2024; Balkhair et al., 2025; Ballard et al., 2024; Guo et al., 2023; Jeznach et al., 2024; Kim et al., 2026; Liu et al., 2025; Stark et al., 2025); patient stratification; implant integration prediction; biomarker identification (Asadi Sarabi et al., 2024; Nosrati and Nosrati, 2023)
5. Scientific communication	Generative AI; LLMs; multimodal generative tools; AI-assisted visualization	Language editing; summarization; drafting support; visualization; dissemination	Enhanced accessibility and clarity; increased efficiency in manuscript preparation	Hallucination; fabricated references; hidden bias; authorship ambiguity; overreliance on automation	Globally, as well as TERM specific: Correcting grammar or suggesting alternate phrasing; cross-checking reference use and relevance, summarizing key and/or complex concepts; identifying gaps in communication; fact checking; formatting according to publishers requirements (Mijatović, 2026; Ng, 2026; van Dis et al., 2023)

### 3.2 Design and decision support (Stage 2)

Once a research gap is identified, the focus shifts toward the strategic planning of experiments and the optimization of materials through systematic material selection, scaffold design, and experimental prioritization. This stage utilizes a transition from language-based generative AI to more specialized predictive modeling, Bayesian optimization, and active learning strategies (Table 2). These tools are employed to synthesize protocols and prioritize specific experimental conditions such as biomaterial composition or scaffold geometry (Ahmed et al., 2024; Guo et al., 2023; Liu et al., 2025; Ramesh et al., 2024; Wu et al., 2024; Zhang et al., 2025), thereby reducing the combinatorial burden of trial-and-error experimentation. Despite their efficiency, the primary risks in this stage involve factual errors and biased search spaces, which may lead to results that lack biological plausibility. Therefore, AI-driven designs must be viewed as candidate hypotheses that require expert evaluation and experimental validation to confirm their feasibility in a biological context. Because AI outputs provide candidate directions rather than evidence of biological validity, establishing scientific knowledge still requires human biological validation, experimental verification, and critical assessment of what is biologically meaningful. Likewise, AI can assist not only in identifying promising experimental conditions but also in extracting biological insight from unexpected or negative experimental outcomes.

### 3.3 Experimental execution and data generation (Stage 3)

As the pipeline moves from design to execution, the technology shift toward computer vision, sensor-based monitoring, and process modeling is critical for real-time quality control (Table 2). AI-based image analysis is now routinely used for tasks such as cell counting, segmentation, and organoid monitoring (Balkhair and Albalushi, 2025; Jeznach et al., 2024; Zhang et al., 2025). Because AI is now participating in the generation of biological evidence, the risk of platform-specific bias, equipment dependency, and model drift becomes a primary translational concern. To mitigate these risks, researchers should implement rigorous calibration protocols and regular sensor validation to ensure that computational representations remain faithful to the direct biological observations. Validation requirements increase substantially when these tools are integrated into feedback, quality control, or release-related workflows. Such tools should function as assistants rather than autonomous controllers, requiring continuous human oversight to identify experimental failure modes early.

### 3.4 Data interpretation and modeling (Stage 4)

The most mature applications of AI in TERM reside in data interpretation, where supervised and unsupervised learning, deep learning, and multimodal learning are used to decode complex biological signatures (Table 2). These tools enable the classification of cell fates, the prediction of biomaterial performance, and the integration of high-dimensional omics data (Ahmed et al., 2024; Balkhair and Albalushi, 2025; Ballard et al., 2024; Guo et al., 2023; Jeznach et al., 2024; Kim et al., 2026; Liu et al., 2025; Stark et al., 2025). Crucially, this stage involves critical preprocessing steps, including noise reduction, clustering, and batch correction, to ensure that downstream interpretation is based on biological signal rather than technical noise.

Despite their predictive power, the “black box” nature of deep learning introduces risks regarding interpretability, overfitting, and batch effects, particularly when working with small, heterogeneous datasets. To mitigate these risks, researchers should prioritize the use of uncertainty quantification and prospective testing on independent cohorts. Ultimately, the goal for the field is to move beyond statistical correlation toward a mechanistic understanding of identified patterns, ensuring that predictive performance is not mistaken for biological causality (Plant et al., 2022; Waters et al., 2021). Thus, high predictive performance does not necessarily imply biological understanding, mechanistic explanation, or causal insight, and these distinctions remain essential for scientific interpretation.

### 3.5 Scientific communication (Stage 5)

The final stage of the pipeline leverages generative AI, LLMs, and multimodal generative tools to streamline the dissemination of TERM research (Table 2). These technologies are routinely used for language editing, summarization, and the preparation of grant applications, protocol documentation, and graphical abstracts (Ng, 2026; van Dis et al., 2023). While these tools enhance accessibility and communication efficiency, their integration into scientific writing introduces substantial risks, including the production of fabricated references, hidden biases, and authorship ambiguity (Cirillo et al., 2020; Ferrara, 2023; Resnik and Hosseini, 2026; Topaz et al., 2026; van Dis et al., 2023). Of particular concern is the capacity of AI platforms to inadvertently incorporate fraudulent, “paper-mill” generated, or retracted literature into knowledge synthesis (Abalkina et al., 2025; Else and Van Noorden, 2021; Kendall and Teixeira da Silva, 2024; Kobak et al., 2025; Thelwall, 2025; Topaz et al., 2026).

To maintain scientific integrity, AI should be employed as an assistive communication technology rather than an independent scientific author or evaluator (Brainard, 2023; Rolauffs et al., 2026; Thorp, 2023; Mijatović, 2026). Methodologically, this requires transparent disclosure of AI assistance and strict adherence to the evolving requirements of publishers (Nature, 2023; Thorp and Vinson, 2023) and funding bodies, including those issued by the NIH, NSF, UKRI, and Wellcome, together with guidance from the European Commission, International Committee of Medical Journal Editors (ICMJE) and other advisory and regulatory guidance organizations (Committee on Publication Ethics (COPE), 2023; European Commission, 2026; European Research Council, 2026; National Institutes of Health, 2025; National Science Foundation, 2023; UK Research and Innovation, 2024; Wellcome Trust, 2026; International Committee of Medical Journal Editors, 2025). Because the nuance of biological context and methodological detail is essential for accurate interpretation, human authors must retain full accountability for the provenance, integrity, and interpretation of all scientific claims.

### 3.6 Translational gaps and future priorities for AI in TERM

Although AI research in TERM is growing rapidly, clinical evidence remains limited. Many studies are still retrospective, single-site, platform-specific, or proof-of-concept. Few studies have shown that model performance is maintained across donors, laboratories, manufacturing settings, or prospective clinical workflows. Encouragingly, several data-sharing initiatives are emerging to address this gap, including the Community Resource for Innovation in Polymer Technology (CRIPT), a repository for polymer science and engineering data (CRIPT, 2026) and BIOMATDB, which catalogues biomaterials metadata such as composition, biocompatibility, performance, and safety data (BIOMATDB, 2026). Evidence linking AI-supported TERM approaches to long-term patient outcomes remains particularly limited, as most current studies are preclinical or rely on short-term surrogate endpoints. Therefore, translational value should not be judged by predictive performance alone. It should also be judged by whether AI improves reproducibility, decision-making, safety, or patient-relevant outcomes in real-world settings (Guo et al., 2023; Klabukov et al., 2026).

AI methods should be selected according to the specific constraints of the task, required interpretability, and intended context of use. Data-driven methods, including deep learning, can detect complex patterns in imaging and omics data and may achieve high predictive performance; however, they often require larger datasets and can be difficult to interpret. In contrast, mechanistic models encode established biological or physical laws, whereas hybrid models combine these constraints with data-driven learning. Both approaches usually require more time and prior knowledge to develop, but can offer greater biological plausibility, support extrapolation, and improve traceability. By adopting this hybrid approach, researchers can leverage the pattern-recognition power of AI while maintaining the safety-guardrails and biological plausibility provided by mechanistic laws. Consequently, deep learning may therefore be suitable for pattern-recognition tasks, whereas hybrid models may be more preferable when biological constraints or mechanistic interpretation are important (Waters et al., 2021; Jeznach et al., 2024).

Systems biology provides a complementary framework for such integrative approaches by contextualizing heterogeneous molecular, cellular, and functional readouts in relation to biological networks and mechanistic relationships (Cadavid et al., 2024; Silva-Sousa et al., 2025). Growing convergence between systems-biology and AI approaches has also been reported in both stem cell research and the development of therapeutics (Silva-Sousa et al., 2024; Silva-Sousa et al., 2025). This may be particularly relevant in TERM, where individual studies are often small and biological information is distributed across different experimental platforms and data modalities. Integrating evidence across such datasets may increase the information available for biological interpretation and model development (Silva-Sousa et al., 2025), but does not by itself resolve the challenges posed by small sample sizes or cross-study heterogeneity. Accordingly, combined systems-biology and AI approaches may be most valuable when integration preserves biological context and explicitly accounts for cross-study and cross-platform variability.

More broadly, future priorities include prospective multi-site validation and standardized benchmark datasets. These datasets should capture variation among donors, batches, platforms, and protocols. Studies should also report uncertainty and failure modes and evaluate AI under manufacturing, clinical, and regulatory conditions (Collins et al., 2024; Liu et al., 2020; Vasey et al., 2022).

## 4 Discussion: Quo vadis? Future directions grounded in purpose and reality

AI is no longer optional for TERM. The central question is how it can be used while preserving scientific rigor, biological judgment, and translational responsibility. This requires purpose-aware AI literacy: researchers must clarify the application domain, define the intended purpose, select an appropriate technology, and understand its associated risks before interpreting outputs or making decisions (European Medicines Agency, 2024; Klabukov et al., 2026; OECD, 2022; Rolauffs et al., 2026). In practice, responsible AI selection could begin by asking four questions: What is the biological question? What decision will the output support? What evidence is required? What level of validation is necessary? Purpose-aware AI use should remain grounded in established scientific standards, while guidance, policy, education, and training evolve with AI. Four principles are central:

- Human-in-the-loop scientific workflows: AI should support experimental reasoning, protocol selection, and interpretation rather than replace human decision-making (Vasey et al., 2022).
- Transparency and traceability: because TERM is closely linked to patients, transparency and traceability are essential responsibilities rather than merely technical requirements. AI-supported findings and decisions should be based on verifiable data, clearly documented assumptions and limitations, and explicit human oversight. Their use should also align with established reproducibility standards and quality control frameworks (National Institute of Standards and Technology, 2023; U.S. Food and Drug Administration, 2023; U.S. Food and Drug Administration, 2025a; U.S. Food and Drug Administration, 2025b; U.S. Food and Drug Administration et al., 2021).
- Acceptable use: researchers need to remain responsible for complying with rapidly evolving community, publisher, and funder requirements concerning AI use, disclosure, and research originality (European Commission, 2026; National Institutes of Health, 2025; National Science Foundation, 2023; UK Research and Innovation, 2024; Wellcome Trust, 2026; International Committee of Medical Journal Editors, 2025).
- Training: educational frameworks must integrate AI not merely as a tool, but as a core competency. This requires a tripartite literacy: biological literacy to formulate meaningful scientific questions, AI literacy to understand system capabilities and limitations, and uncertainty literacy to critically evaluate evidence quality and model confidence. Because generative AI constructs responses rather than simply retrieving data, these competencies—combining technical knowledge with scientific judgment—are essential for the next-generation of TERM researchers to critically evaluate AI-generated outputs and maintain technical discipline (Plant et al., 2022; Tankelevitch et al., 2024).

Parallel to purpose-aware literacy, several methodological competencies are essential for responsible AI integration in TERM:

- Small-n-aware modeling: AI methods should be designed for small, heterogeneous, donor-dependent, and batch-sensitive datasets rather than assuming large, standardized datasets (Gharibshahian et al., 2024; Guo et al., 2023; Klabukov et al., 2026; Plant et al., 2022; Waters et al., 2021).
- Robustness and sensitivity analysis: ideally, models should be tested across donors, batches, platforms, laboratories, imaging conditions, and protocol variations (Collins et al., 2024; Plant et al., 2022).
- Uncertainty quantification: AI outputs should communicate confidence, uncertainty, and possible failure modes rather than only deterministic point predictions (Collins et al., 2024; National Institute of Standards and Technology, 2023; U.S. Food and Drug Administration, 2025b).
- Translation-facing validation: AI models should be evaluated in relation to manufacturing constraints, quality control, traceability, documentation, and regulatory expectations (National Institute of Standards and Technology, 2023; U.S. Food and Drug Administration, 2025a; U.S. Food and Drug Administration, 2025b; U.S. Food and Drug Administration et al., 2021).
- Bias and heterogeneity assessment: donor characteristics, tissue source, disease state, sex, age, and other sources of biological variation should be considered not only as noise but also as potentially meaningful data (Cirillo et al., 2020; Ferrara, 2023; Gharibshahian et al., 2024; Plant et al., 2022).
- Prospective or predefined validation: models should be tested on newly generated data or under predefined evaluation protocols to reduce overfitting to datasets, workflows, or scientific narratives (Collins et al., 2024; Liu et al., 2020; Vasey et al., 2022).

Ultimately, AI does not redefine the scientific method in TERM; rather, it amplifies both the strengths and limitations of existing experimental and translational practices. Scientific discovery remains a human-led process grounded in biological interpretation, experimental rigor, and ethical responsibility. Many critical gaps arise not from algorithms themselves, but from insufficient experimental design, validation frameworks, and education for integrating AI into biological research. Progress will therefore depend on using AI for support rather than substitution, treating its outputs as inputs for expert reasoning rather than final answers, and clearly documenting uncertainty and human accountability. The field’s future will be determined less by algorithmic intelligence than by how responsibly and intelligently researchers integrate AI into scientific practice (Ng, 2026; OECD, 2022; Plant et al., 2022; Resnik and Hosseini, 2025; Rolauffs et al., 2026). The future of AI in TERM will depend less on building increasingly capable algorithms than on developing increasingly capable scientists who know when, why, and how to use them.

## References

[B1] Abalkina A. Aquarius R. Bik E. Bimler D. Bishop D. Byrne J. (2025). ‘Stamp out paper mills’—science sleuths on how to fight fake research. Nature 637, 1047–1050. 10.1038/d41586-025-00212-1 39870791

[B2] Ahmed E. Mulay P. Ramirez C. Tirado-Mansilla G. Cheong E. Gormley A. J. (2024). Mapping biomaterial complexity by machine learning. Tissue Eng. Part A 30, 662–680. 10.1089/ten.TEA.2024.0067 39135398 PMC12394815

[B3] American Society of Mechanical Engineers (2018). “Assessing credibility of computational modeling through verification and validation: application to medical devices”. ASME V&V 40-2018. New York, NY: ASME.

[B4] Asadi Sarabi P. Shabanpouremam M. Eghtedari A. R. Barat M. Moshiri B. Zarrabi A. (2024). AI-based solutions for current challenges in regenerative medicine. Eur. J. Pharmacol. 984, 177067. 10.1016/j.ejphar.2024.177067 39454850

[B5] Balkhair O. Albalushi H. (2025). Artificial intelligence in organoid-based disease modeling: a new frontier in precision medicine. Biomimetics 10, 845. 10.3390/biomimetics10120845 41439914 PMC12730694

[B6] Ballard J. L. Wang Z. Li W. Shen L. Long Q. (2024). Deep learning-based approaches for multi-omics data integration and analysis. BioData Min. 17, 38. 10.1186/s13040-024-00391-z 39358793 PMC11446004

[B7] Banerjee S. Lahiri S. K. (2026). Digital evolution of bioreactor's trends and frontiers in artificial intelligence/machine learning-driven process intelligence. Syst. Microbiol. Biomanuf. 6, 71. 10.1007/s43393-026-00460-w

[B8] BIOMATDB (2026). Advanced database for biomaterials with data analysis and visualisation tools. Available online at: https://www.biomatdb.eu (Accessed July 30, 2026).

[B9] Brainard J. (2023). As scientists explore AI-written text, journals hammer out policies. Science 379, 740–741. 10.1126/science.adh2762 36821673

[B10] Cadavid J. L. Li N. T. McGuigan A. P. (2024). Bridging systems biology and tissue engineering: unleashing the full potential of complex 3D *in vitro* tissue models of disease. Biophys. Rev. 5, 021301. 10.1063/5.0179125 38617201 PMC11008916

[B11] Cirillo D. Catuara-Solarz S. Morey C. Guney E. Subirats L. Mellino S. (2020). Sex and gender differences and biases in artificial intelligence for biomedicine and healthcare. NPJ Digit. Med. 3, 81. 10.1038/s41746-020-0288-5 32529043 PMC7264169

[B12] Collins G. S. Moons K. G. M. Dhiman P. Riley R. D. Beam A. L. Van Calster B. (2024). TRIPOD+AI statement: updated guidance for reporting clinical prediction models that use regression or machine learning methods. BMJ 385, e078378. 10.1136/bmj-2023-078378 38626948 PMC11019967

[B68] Committee on Publication Ethics (COPE) (2023). Authorship and AI Tools. Available online at: https://publicationethics.org/guidance/cope-position/authorship-and-ai-tools (Accessed August 21, 2026).

[B13] CRIPT (2026). A community resource for innovation in polymer technology. Available online at: https://www.criptapp.org/(Accessed July 30, 2026). 10.1021/acscentsci.3c00011 PMC10037456 36968543

[B14] Else H. Van Noorden R. (2021). The fight against fake-paper factories that churn out sham science. Nature 591, 516–519. 10.1038/d41586-021-00733-5 33758408

[B15] European Commission (2026). Living guidelines on the responsible use of generative AI in research. ERA Forum Stakeholders’ Doc. Third version, May 2026. Available online at: https://research-and-innovation.ec.europa.eu/document/download/2b6cf7e5-36ac-41cb-aab5-0d32050143dc_en?filename=ec_rtd_ai-guidelines.pdf .

[B16] European Medicines Agency (2024). Reflection Paper on the Use of Artificial Intelligence in the Medicinal Product Lifecycle. EMA/CHMP/CVMP/83833/2023.

[B17] European Research Council (ERC) (2026). The Use of AI in Grant Proposal Evaluation: Guidelines for ERC Panel Members and Remote Reviewers. Available online at: https://erc.europa.eu/system/files/2026-03/Use-AI-grant-proposal-evaluation.pdf (Accessed July 30, 2026).

[B18] Ferrara E. (2023). Fairness and bias in artificial intelligence: a brief survey of sources, impacts, and mitigation strategies. Sci 6 (1), 3. 10.3390/sci6010003

[B19] Garmany A. Terzic A. (2024). Artificial intelligence powers regenerative medicine into predictive realm. Regen. Med. 19, 611–616. 10.1080/17460751.2024.2437281 39660914 PMC11703382

[B20] Gharibshahian M. Torkashvand M. Bavisi M. Aldaghi N. Alizadeh A. (2024). Recent advances in artificial intelligent strategies for tissue engineering and regenerative medicine. Skin. Res. Technol. 30, e70016. 10.1111/srt.70016 39189880 PMC11348508

[B21] Guo J. L. Januszyk M. Longaker M. T. (2023). Machine learning in tissue engineering. Tissue Eng. Part A 29, 2–19. 10.1089/ten.TEA.2022.0128 35943870 PMC9885550

[B22] Helleckes L. M. Putz S. Gupta K. Franzreb M. Garcia Martin H. (2026). Perspectives for artificial intelligence in bioprocess automation. Curr. Opin. Biotechnol. 97, 103392. 10.1016/j.copbio.2025.103392 41317522

[B23] International Committee of Medical Journal Editors (2025). Recommendations for the conduct, reporting, editing, and publication of scholarly work in medical journals. Available online at: https://www.icmje.org/recommendations/(Accessed July 5, 2026). 10.12771/emj.2024.e48 PMC12093628 40704003

[B24] Jeznach O. Tabakoglu S. Zaszczyńska A. Sajkiewicz P. (2024). Review on machine learning application in tissue engineering: what has been done so far? Application areas, challenges, and perspectives. J. Mat. Sci. 59, 21222–21250. 10.1007/s10853-024-10449-2

[B26] Kendall G. Teixeira da Silva J. A. (2024). Risks of abuse of large language models, like ChatGPT, in scientific publishing: authorship, predatory publishing, and paper mills. Learn. Publ. 37, 55–62. 10.1002/leap.1578

[B27] Kim D. Youn J. Kim J. Lee J. Yoon J. Kim D. S. (2026). From organoid culture to manufacturing: technologies for reproducible and scalable organoid production. NPJ Biomed. Innov. 3, 12. 10.1038/s44385-025-00054-6 42031991 PMC13042217

[B28] Klabukov I. Skornyakova E. Potievskiy M. Atiakshin D. Sulina Y. Yakimova A. (2026). Limitations of artificial intelligence in tissue engineering and regenerative medicine. Biomed. Res. Ther. 13, 8193–8202. 10.15419/4k8jf512

[B29] Kobak D. González-Márquez R. Horvát E.-Á. Lause J. (2025). Delving into LLM-assisted writing in biomedical publications through excess vocabulary. Sci. Adv. 11, eadt3813. 10.1126/sciadv.adt3813 40601754 PMC12219543

[B30] Liu X. Rivera S. C. Moher D. Calvert M. J. Denniston A. K. , and CONSORT-AI (2020). Reporting guidelines for clinical trial reports for interventions involving artificial intelligence: the CONSORT-AI extension. Nat. Med. 26, 1364–1374. 10.1038/s41591-020-1034-x 32908283 PMC7598943

[B31] Liu M. Zhou Y. Mei X. Yu Z. Guan B. Xiao Y. (2025). AI-driven biomaterial design: an intelligent closed loop from reverse design to biological response. Front. Cell Dev. Biol. 13, 1755565. 10.3389/fcell.2025.1755565 41561627 PMC12813142

[B32] Mijatović A. Žuljević M. F. Ursić L. Marušić A. (2026). Responsible use of large language models in manuscript preparation. Curr. Protoc. 6, e70300. 10.1002/cpz1.70300 41511390

[B33] Moroni L. Burdick J. A. Highley C. Lee S. J. Morimoto Y. Takeuchi S. (2018). Biofabrication strategies for 3D *in vitro* models and regenerative medicine. Nat. Rev. Mat. 3, 21–37. 10.1038/s41578-018-0006-y 31223488 PMC6586020

[B34] National Institute of Standards and Technology (2023). “Artificial intelligence risk management framework (AI RMF 1.0),”. NIST AI 100-1. Gaithersburg, MD: National Institute of Standards and Technology. 10.6028/NIST.AI.100-1

[B35] National Institutes of Health (2025). NOT-OD-25-132: supporting fairness and originality in NIH research applications. Available online at: https://grants.nih.gov/grants/guide/notice-files/NOT-OD-25-132.html (Accessed July 5, 2026).

[B36] National Science Foundation (2023). Notice to the research community: use of generative AI technology in the NSF merit review process. Available online at: https://www.nsf.gov/news/notice-to-the-research-community-on-ai (Accessed July 5, 2026).

[B37] Nature (2023). Tools such as ChatGPT threaten transparent science;here are our ground rules for their use. Nature 613, 612. 10.1038/d41586-023-00191-1 36694020

[B38] Ng J. Y. (2026). A structured framework for effective and responsible generative artificial intelligence chatbot prompt engineering throughout the scientific process: a comprehensive guide for the health and medical researcher. Front. Artif. Intell. 9, 1745928. 10.3389/frai.2026.1745928 42080052 PMC13130486

[B39] Nosrati H. Nosrati M. (2023). Artificial intelligence in regenerative medicine: applications and implications. Biomimetics 8, 442. 10.3390/biomimetics8050442 37754193 PMC10526210

[B40] OECD (2022). “OECD framework for the classification of AI systems,”. OECD Digital Economy Papers, No. 323. Paris: OECD Publishing. 10.1787/cb6d9eca-en

[B41] Plant A. L. Piscopo N. Saha K. Zylberberg C. Roy K. Tsokas K. (2022). Implementing systems thinking and data science in the training of the regenerative medicine workforce. NPJ Regen. Med. 7, 76. 10.1038/s41536-022-00271-2 36566283 PMC9790008

[B42] Ramesh S. Deep A. Tamayol A. Kamaraj A. Mahajan C. Madihally S. (2024). Advancing 3D bioprinting through machine learning and artificial intelligence. Bioprinting 38, e00331. 10.1016/j.bprint.2024.e00331

[B44] Resnik D. B. Hosseini M. (2025). The ethics of using artificial intelligence in scientific research: new guidance needed for a new tool. AI Ethics.5:1499–1521. 10.1007/s43681-024-00493-8 40337745 PMC12057767

[B45] Resnik D. B. Hosseini M. (2026). Hallucinated citations produced by generative artificial intelligence may constitute research misconduct when citations function as data in scholarly papers. Acc. Res. 2645390. 10.1080/08989621.2026.2645390 41833014 PMC13051339

[B46] Rolauffs B. Kato R. Hart M. L. Raimondo C. Walsh M. C. (2026). Rethinking the scientific method in the age of AI. Nat. Rev. Bioeng. 4, 2–3. 10.1038/s44222-025-00386-3

[B47] Silva-Sousa T. Usuda J. N. Al-Arawe N. Frias F. Hinterseher I. Catar R. (2024). The global evolution and impact of systems biology and artificial intelligence in stem cell research and therapeutics development: a scoping review. Stem Cells 42, 929–944. 10.1093/stmcls/sxae054 39230167

[B48] Silva-Sousa T. Usuda J. N. Al-Arawe N. Hinterseher I. Catar R. Luecht C. (2025). Artificial intelligence and systems biology analysis in stem cell research and therapeutics development. Stem Cells Transl. Med. 14, szaf037. 10.1093/stcltm/szaf037 41015943 PMC12476622

[B49] Song P. Wu Y. Zhang C. Zhou F. Bai L. Su J. (2025). Materiobiology in the omics era. Mater Today Bio 35, 102539. 10.1016/j.mtbio.2025.102539 41332940 PMC12666584

[B50] Stark C. Jenison S. E. Ngo M. T. (2025). -Omics approaches to study and model cell-cell interactions in engineered tissues. Front. Chem. Eng. 7, 1629455. 10.3389/fceng.2025.1629455

[B51] Tankelevitch L. Kewenig V. Simkute A. Scott A. E. Sarkar A. Sellen A. (2024). “The metacognitive demands and opportunities of generative AI,” in Proceedings of the 2024 CHI Conference on Human Factors in Computing Systems. New York, NY: Association for Computing Machinery, 1–24.

[B52] Thelwall M. Lehtisaari M. Katsirea I. Holmberg K. Zheng E. (2025). Does Chatgpt Ignore Article Retractions and Other Reliability Concerns? Learn. Publ. 10.1002/leap.2018

[B53] Thorp H. H. (2023). ChatGPT is fun, but not an author. Science 379, 313. 10.1126/science.adg7879 36701446

[B54] Thorp H. H. Vinson V. (2023). Change to policy on the use of generative AI and large language models. Science. Available online at: https://www.science.org/content/blog-post/change-policy-use-generative-ai-and-large-language-models (Accessed September 14, 2026).

[B55] Topaz M. Roguin N. Gupta P. Zhang Z. Peltonen L. M. (2026). Fabricated citations: an audit across 2.5 million biomedical papers. Lancet 407, 1779–1781. 10.1016/S0140-6736(26)00603-3 42107362

[B56] UK Research and Innovation (2024). Generative artificial intelligence in application and assessment policy. Available online at: https://www.ukri.org/publications/generative-artificial-intelligence-in-application-and-assessment-policy/(Accessed July 5, 2026).

[B57] U.S. Food and Drug Administration (2023). Assessing the Credibility of Computational Modeling and Simulation in Medical Device Submissions: Guidance for Industry and Food and Drug Administration Staff. Silver Spring, MD: U.S. Food and Drug Administration. Available online at: https://www.fda.gov/regulatory-information/search-fda-guidance-documents/assessing-credibility-computational-modeling-and-simulation-medical-device-submissions (Accessed July 7, 2026).

[B58] U.S. Food and Drug Administration (2025a). Artificial Intelligence and Machine Learning (AI/ML) for Biological and Other Products Regulated by CBER. Silver Spring, MD: Center for Biologics Evaluation and Research. Available online at: https://www.fda.gov/vaccines-blood-biologics/artificial-intelligence-and-machine-learning-aiml-biological-and-other-products-regulated-cber (Accessed July 5, 2026).

[B59] U.S. Food and Drug Administration (2025b). Considerations for the Use of Artificial Intelligence to Support Regulatory Decision-Making for Drug and Biological Products: Draft Guidance for Industry and Other Interested Parties. Docket No. FDA-2024-D-4689. Silver Spring, MD: U.S. Food and Drug Administration. Available online at: https://www.fda.gov/regulatory-information/search-fda-guidance-documents/considerations-use-artificial-intelligence-support-regulatory-decision-making-drug-and-biological (Accessed July 5, 2026).

[B60] U.S. Food and Drug AdministrationHealth Canadaand Medicines and Healthcare Products Regulatory Agency (2021). Good Machine Learning Practice for Medical Device Development: Guiding Principles. Available online at: https://www.fda.gov/medical-devices/software-medical-device-samd/good-machine-learning-practice-medical-device-development-guiding-principles (Accessed July 5, 2026).

[B61] van Dis E. A. M. Bollen J. Zuidema W. van Rooij R. Bockting C. L. (2023). ChatGPT: five priorities for research. Nature 614, 224–226. 10.1038/d41586-023-00288-7 36737653

[B62] Vasey B. Nagendran M. Campbell B. Clifton D. A. Collins G. S. Denaxas S. (2022). Reporting guideline for the early-stage clinical evaluation of decision support systems driven by artificial intelligence: DECIDE-AI. Nat. Med. 28, 924–933. 10.1038/s41591-022-01772-9 35585198

[B63] Vilakati S. (2025). Prompt engineering for accurate statistical reasoning with large language models in medical research. Front. Artif. Intell. 8, 1658316. 10.3389/frai.2025.1658316 41159127 PMC12554733

[B64] Waters S. L. Schumacher L. J. El Haj A. J. (2021). Regenerative medicine meets mathematical modelling: developing symbiotic relationships. NPJ Regen. Med. 6, 24. 10.1038/s41536-021-00134-2 33846347 PMC8042047

[B65] Wellcome Trust (2026). Use of generative AI. Available online at: https://wellcome.org/research-funding/guidance/policies-grant-conditions/use-of-generative-ai (Accessed July 5, 2026).

[B66] Wu Y. Ding X. Wang Y. Ouyang D. (2024). Harnessing the power of machine learning into tissue engineering: current progress and future prospects. Burns Trauma 12, tkae053. 10.1093/burnst/tkae053 39659561 PMC11630859

[B67] Zhang Z. Zhou X. Fang Y. Xiong Z. Zhang T. (2025). AI-driven 3D bioprinting for regenerative medicine: from bench to bedside. Bioact. Mat. 45, 201–230. 10.1016/j.bioactmat.2024.11.021 39651398 PMC11625302

